# Different patterns of longitudinal changes in antinuclear antibodies titers in children with systemic lupus erythematosus and Sjögren's syndrome

**DOI:** 10.1177/09612033241298729

**Published:** 2024-11-04

**Authors:** Patricia Morán Álvarez, Claudia Bracaglia, Rebecca Nicolai, Luigi Giovannelli, Ivan Caiello, Alessandra Boni, Valentina Matteo, Gian Marco Moneta, Virginia Messia, Fabrizio De Benedetti, Emiliano Marasco

**Affiliations:** 1 Division of Rheumatology, Bambino Gesù Children’s Hospital, IRCCS, Rome, Italy; 2 Laboratory of Autoimmunity, Bambino Gesù Children’s Hospital, IRCCS, Rome, Italy; 3 Laboratory of Immuno-Rheumatology, Bambino Gesù Children’s Hospital, IRCCS, Rome, Italy

**Keywords:** Antinuclear antibodies (ANA), extractable nuclear antigen (ENA), sjögren’s syndrome (SS), systemic lupus erythematosus (SLE), interferon (IFN) signature

## Abstract

**Objective:**

to investigate the trend of autoantibody titers during a 2-year follow-up in pediatric systemic lupus erythematosus (pSLE) and pediatric Sjögren's syndrome (pSS).

**Methods:**

Autoantibodies testing was performed every 3-4 months during 2 years from disease onset in a cohort of children with pSLE and pSS.

**Results:**

We enrolled 21 children with pSLE and 22 children with pSS. All pSLE patients at 2 years showed ANA titers significantly lower compared to disease onset. Eleven patients (73%) were still ANA positive at 2 years, while 4 (26%) became ANA negative. At diagnosis, 12 (80%) patients showed a homogeneous pattern, while 3 (20%) patients showed a speckled pattern. The latter remained ANA positive with the same pattern; only 2 patients with a homogenous pattern converted to speckled, 4 patients with a homogeneous pattern became ANA negative. ANA negative pSLE patients showed lower levels of interferon score compared to ANA positive patients. Anti-dsDNA titers declined equally in the two groups. All patients with pSS, at disease onset, were ANA and anti-Ro positive and 14 (66%) were anti-La positive. After 2 years of follow-up, 100% remained ANA positive but showed significant lower titers. During follow-up anti-Ro and anti-La titers remained stable.

**Conclusion:**

different patterns in changes of ANA and ENA titers in pSLE and pSS were shown. At 2 years of follow-up, all pSLE patients had a lower ANA titer and 26% became negative; however, all pSS patients remained both ANA and ENA positive. This evidence may be due to different pathogenetic pathways in SLE and pSS.

## Introduction

Systemic lupus erythematosus (SLE) is a chronic, life-threatening, and multi-systemic autoimmune disease, whose hallmark feature is the presence of antinuclear antibodies (ANA). These antibodies bind to specific antigens with the formation of immune complexes and their deposition in tissues, leading to inflammation and organ damage. Some ANA bind double strand DNA or associated nucleosome proteins; whereas, other ANA bind protein components of complexes of RNA and RNA-binding proteins (RBPs), also known as extractable nuclear antigen (ENA). According to the 2019 European Alliance of Associations for Rheumatology/ American College of Rheumatology (EULAR/ACR) SLE classification criteria,^1^ a positive ANA test is a mandatory item to classify patients as having SLE. ANA positivity is defined as an immunofluorescence assay (IFA) at a titer of ≥1:80 on Hep-2 cells or an equivalent positive test if other assays have been used, even though a precise definition of these equivalences have not yet been specified. ANA IFA continues to be the ‘gold-standard’ screening test; however, recent studies have demonstrated that the combination of at least two different assays are a useful strategy to reduce the proportion of SLE patients with a negative ANA test. In clinical trials up to 30% of patients with an established diagnosis of SLE were found to be ANA negative at enrollment.^2^ This observation may be related to differences in assay performance, but, at the same time, it may be the result of other factors, such as the demographic characteristics of patients (i.e., race or age), the natural history of the disease or the effects of treatments.

Sjögren’s syndrome (SS) is a multisystem autoimmune disease characterized by inflammation of salivary and lacrimal glands, with varying degrees of systemic involvement. Similarly to SLE, SS is characterized by the presence of ANA and ENA, specifically anti-Ro and-La autoantibodies. According to the 2016 ACR-EULAR Classification Criteria for Sjögren’s Syndrome, patients complaining with one symptom of ocular or oral dryness, can be classified as having SS if they reach a score of 4 based on the weighted sum of five items. Of these items, anti-Ro positivity confers three points, the same weight as observing lymphocytic sialadenitis on labial salivary gland biopsy, highlighting the importance of anti-Ro positivity in the diagnosis of SS. Only few studies investigated the longitudinal changes of anti-Ro and anti-La antibodies, without observing a consistent pattern: most of patients showed fluctuating levels of autoantibodies over time, without clear association with disease activity.^3,4^

The primary objective of our study was to investigate the trend of autoantibodies titers and pattern over time, during a 2-year follow-up, in children with a diagnosis of pediatric onset systemic lupus erythematosus (pSLE) and pediatric Sjögren’s Syndrome (pSS).

## Methods

### Study design

We performed an historical cohort study of children with pSLE and pSS to determine the trend over time of ANA, anti-dsDNA and ENA.

### Study population

We enrolled 21 children with a diagnosis of pSLE with disease onset under the age of 18 years, followed at an Italian tertiary hospital (Ospedale Pediatrico Bambino Gesù). Patients fulfilled the 2019 EULAR/ACR SLE classification criteria. For 15 children with pSLE we had data at disease onset. The remaining 6 patients were characterized at 2-year follow-up as described in the results section.

We also enrolled 22 children with a diagnosis of pSS based on the proposed criteria by Bartunkova, et al.^5^ Consistent with the literature, seven patients (31.8%) met the 2016 ACR/EULAR criteria for SS.^6^ In our study we included only patients whose autoantibody determinations had been performed at our hospital at the disease-onset before the initiation of any treatment, and afterwards every 3-4 months during the 2-year follow-up.

The protocol was approved by the local Ethics Committee for Clinical Research at our hospital (1666_OPBG_2018); the study was performed following the principles outlined in the Helsinki Declaration. Written informed consent was obtained by all patients or their parents in case of age under 18 years.

We excluded from this longitudinal study children who: (i) did not fulfill the 2019 EULAR/ACR SLE classification criteria; (ii) did not fulfill the proposed preliminary pSS criteria (iii), (iv) had not been followed from disease onset; (v) had been under immunosuppressive therapy prior to the first autoantibody panel determination; or (vi) had not completed the 2 years of follow-up.

Clinical and laboratory data were retrieved from electronic records. For SLE, disease activity was assessed by SLEDAI in the 10 days preceding ANA evaluation. For pSS, disease activity was assessed with the EULAR Sjogren’s syndrome disease activity index (ESSDAI).

### Autoantibody testing

Sera were obtained every 3-4 months during 2 years of follow up from disease onset. ANA test was performed using a commercial immunoflorescence assay (IFA) (INOVA diagnostics, Inc.). Slides with fixed HEp-2 cells were used as antigen substrate and incubated with diluted serum according to manufacturer’s instructions. Fluorescein isothiocyanate (FITC)-conjugated anti-human immunoglobulin G was used as detection antibody. Serum samples were first tested at a dilution of 1:80 in phosphate buffered saline (PBS). Positive samples were titrated in twofold dilutions steps starting from 1:40 up to 1:20480. Positive ANA tests were categorized regarding staining patterns according to the International Consensus of ANA Patterns (ICAP) nomenclature.^7^

Anti-dsDNA antibodies were tested using a commercial immunofluorescence assay (IFA) (INOVA diagnostics, Inc.). DNA of the kinetoplast of Crithidia luciliae was used as antigen substrate and incubated with diluted serum according to manufacturer’s instructions. Fluorescein isothiocyanate (FITC)-conjugated anti-human immunoglobulin G/M/A was used as detection antibody in the screening test. Serum samples were first tested at a dilution of 1:10 in phosphate buffered saline (PBS). Positive samples were characterized with monospecific secondary antibodies against IgG or IgM or IgA (Euroimmun) and then titrated in twofold dilutions steps starting from 1:10 up to 1:20480.

Autoantibodies against Extractable Nuclear Antigen (Ro, La, Sm, U1-RNP, Jo1, CENP, Scl70) were tested with a quantitative automated solid-phase fluorescence enzyme immunoassays (FEIA) (Phadia Laboratory Systems) according to manufacturer’s instructions.

### Interferon score

Whole blood from controls (*n* = 10) and patients with pSLE (*n* = 21) was collected into PAXgene tubes (PreAnalytix) at 2 years after diagnosis. Whole blood total RNA was extracted with a PAXgene RNA isolation kit (PreAnalytix). Quantification of RNA and assessment of RNA purity were conducted by spectrophotometry (NanoDrop 2000; Thermo Fisher). To assess the integrity of RNA, samples were run on agarose gel. For each sample 2.5 µg of total RNA was reverse transcribed using a Superscript VILO-cDNA-synthesis Kit (Invitrogen). Gene expression was measured by quantitative PCR with the following probes: IFI27, Hs01086370_m1; IFI44 L, Hs00199115_m1; IFIT1, Hs00356631_g1; ISG15, Hs00192713_m1; RSAD2, Hs01057264_m1; GAPDH, Hs99999905_m1 (Applied Biosystems). Gene expression data were normalized using GAPDH as endogenous control. Quantitative PCR reactions were performed on the 7900-HT-Fast-Real-Time-PCR System, using Taqman universal Master Mix (Applied-Biosystems, Foster-City, CA). Fold changes were calculated using the 2^−ΔΔCt^ equation using controls as reference. As previously described by Rice *et al*,^8^ median fold change of type I IFN-inducible genes, compared with median of combined controls, was used to calculate the type I IFN score for each patient.

### Statistical analysis

A descriptive analysis was performed for demographic, clinical and laboratory variables. Numerical variables were described using mean and standard deviation (SD) or median and interquartile range (IQR). Absolute and relative frequencies were calculated for qualitative variables.

A bivariate analysis was conducted; qualitative variables were assessed by χ^2^, Yates correction, Fisher exact or McNemar’s tests in 2 × 2 tables, according to the distribution of each variable. Numerical variables were assessed using the Mann-Whitney *U* for independent samples and *Wilcoxon* test for paired samples respectively. All analysis were performed using R software, version 4.0.3. A *p-value* less than 0.05 was considered statistically significant.

## Results

### Autoantibodies in patients with pSLE

**Figure 1.**
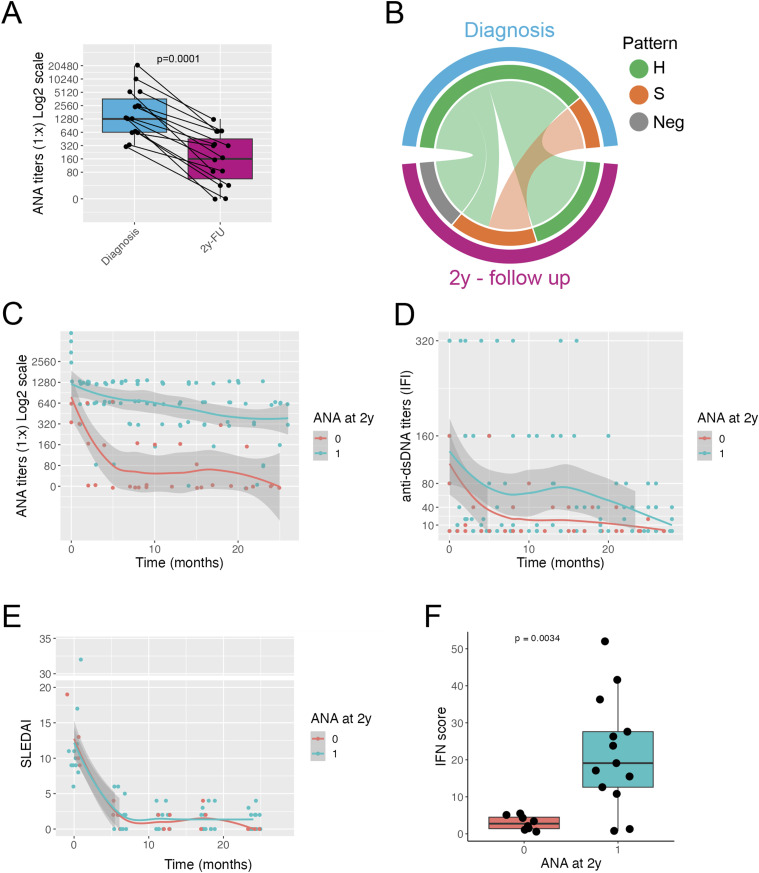
Autoantibody profile in patients with pSLE. Titers of ANA at diagnosis and at 2-years follow-up; connected dots indicate individual patients; red dots indicate patients that become ANA negative at 2 years follow-up (a). Circos plot showing the frequency of ANA pattern at diagnosis and at 2-years follow-up (b). Plot showing the ANA titers at diagnosis and over a 2-years follow-up; lines indicate average trend with shaded 95% confidence intervals for the two groups Two groups defined according to ANA status at 2-years follow-up: Group 0. ANA neg and Group 1: ANA pos) (c). Plot showing the anti-dsDNA titers at diagnosis and over a 2-years follow-up time; lines indicate average trend with shaded confidence intervals for the two groups. Two groups defined as in (c) (d). Plot showing SLEDAI score at diagnosis and over a 2-years follow-up time; lines indicate average trend with shaded confidence intervals for the two groups, defined as in panel (c) (e). Interferon score at 2 years after diagnosis for the two groups, defined as in panel (c) (f). A, Wilcoxon test; F, Mann–Whitney U test. H = homogenous pattern, S = speckled pattern, Neg = negative ANA test.

A total of 15 children with a diagnosis of pSLE fulfilling the 2019 EULAR/ACR SLE classification criteria were enrolled in the study for the longitudinal analysis of the autoantibody profile. They were followed every 3-4 months for 2 years from the disease onset. The demographic, clinical and laboratory features of patients with pSLE are shown in Table 1. Upon diagnosis, all patients were started on glucocorticoids, hydroxychloroquine and mycophenolate. Additionally, two patients received rituximab. After a 2-year follow-up, the treatment regimen remained unchanged (Table 1). All patients had high titers of ANA at diagnosis. Only four patients were positive for ENAs (2 anti-Sm, 1 anti-RNP and 1 anti-Ro). All patients at 2-year follow-up showed ANA titers significantly lower than at time of SLE diagnosis (Wilcoxon test, *p* = .0001) (Figure 1(a)). At diagnosis 12 (80%) patients showed a homogeneous pattern, the remaining three (20%) patients showed a speckled pattern (Figure 1(b)). The ANA pattern remained fairly constant during follow-up: all patients with a speckled pattern remained ANA positive with a speckled pattern; only two patients with a homogenous ANA pattern converted to a speckled pattern after 2 years, four patients with a homogeneous pattern became ANA negative (Figure 1(b)).

**Table 1.** Demographic and clinical characteristics at diagnosis and therapy at 2-years follow up of 15 patients with pSLE divided based on ANA positivity at 2-years follow up (ANA negative, Group 0; ANA positive, Group 1).

	All *n* = 15	Group 0 *n* = 4	Group 1 *n* = 11	*p*-value
Age at diagnosis, years [median, IQR]	12.63 [12.24, 14.84]	13.00 [12.33, 14.81]	12.63 [12.20, 14.54]	0.514
Female patients, *n* (%)	13 (87.0)	3 (75.0)	10 (90.9)	1.000
SLEDAI-2K at diagnosis [median, IQR]	10.00 [9.00, 12.50]	12.00 [9.75, 14.50]	10.00 [9.00, 11.50]	0.428
SLEDAI-2K at follow-up [median, IQR]	0.00 [0.00, 2.00]	0.00 [0.00, 0.00]	2.00 [0.00, 2.00]	0.074
LLDAS at follow-up, *n* (%)	9 (60)	3 (75)	6 (54)	0.600
DORIS remission at follow-up, *n* (%)	2 (15)	1 (25)	1 (9)	0.540
C3, mg/dL [median, IQR]	45.00 [36.00, 65.00]	40.0 [37.00, 42.50]	60.00 [37.50, 68.75]	0.310
C4, mg/dL [median, IQR]	4.00 [2.00, 6.00]	2.0 [2.00, 4.50]	4.00 [2.50, 6.00]	0.666
Autoantibody profile				
anti-Sm+, *n* (%)	2 (13.3)	1 (25.0)	1 (9.0)	NA
anti-RNP+, *n* (%)	1 (6.6)	0 (0.0)	1 (9.0)	NA
anti-Ro+, *n* (%)	1 (6.6)	0 (0.0)	1 (9.0)	NA
anti-La+, *n* (%)	0 (0.0)	0 (0.0)	0 (0.0)	NA
LAC, *n* (%)	1 (6.7)	0 (0.0)	1 (9.1)	NA
anti-beta2GPI IgM+, *n* (%)	2 (13.3)	1 (25.0)	1 (9.0)	0.476
anti-beta2GPI IgG+, *n* (%)	2 (13.3)	1 (25)	1 (9.0)	0.476
anti-Cardiolipin IgM+, *n* (%)	2 (13.3)	1 (25)	1 (9.0)	0.476
anti-Cardiolipin IgG+, *n* (%)	2 (13.3)	1 (25.0)	1 (9.0)	0.476
anti-dsDNA + at diagnosis, *n* (%)	12 (80.0)	3 (75.0)	9 (81.0)	1.000
anti-dsDNA + at follow-up, *n* (%)	7 (46.6)	1 (25.0)	6 (54.5)	0.569
Clinical features				
Mucocutaneous, *n* (%)	10 (66.7)	4 (100.0)	6 (54.5)	0.302
Serositis, *n* (%)	2 (13.3)	0 (0.0)	2 (18.2)	0.954
Heamatological, *n* (%)	14 (93.3)	4 (100.0)	10 (90.9)	1.000
Musculoskeletal, *n* (%)	7 (46.7)	3 (75.0)	4 (36.4)	0.459
Constitutional, *n* (%)	11 (73.3)	3 (75.0)	8 (72.7)	1.000
Lupus nephritis, *n* (%)^a^	3 (20.0)	1 (25.0)	2 (18.2)	1.000
Medications during 2-year follow-up				
Glucocorticoids, *n* (%)	14 (93.3)	3 (75.0)	11 (100.0)	0.585
Glucocorticoid dose, mg/die	7.5 [6.2, 10.0]	7.5 [6.2, 8.1]	7.5 [6.2, 10.0]	0.496
Hydroxychloroquine, *n* (%)	15 (100.0)	4 (100.0)	11 (100.0)	NA
Mycophenolate mofetil, *n* (%)	15 (100.0)	4 (100.0)	11 (100.0)	NA
Rituximab, *n* (%)	2 (13.3)	0 (0.0)	2 (18.2)	0.954
Cyclophosphamide, *n* (%)	0 (0)	0 (0)	0 (0)	NA

^a^All patients with LN had a class III nephritis.

Abbreviations: LLDAS, Lupus low disease activity; DORIS, definition Of Remission In SLE.

After 2 years of follow-up, 11 patients (73%) still showed positive ANA (Group 1), while four patients (26%) became ANA negative (Group 0). Assessing the change over time in ANA titers, the two groups of patients showed two different patterns: in Group 0, ANA titers quickly declined and became negative in the first 6 months after diagnosis of pSLE; in Group 1, ANA titers declined more slowly, remaining positive throughout the follow-up (Figure 1(c)). Anti-dsDNA antibodies titers declined over time with no clear different patterns between the two groups (Figure 1(d), Supplemental Figure 1(A)). C3 and C4 levels increased significantly during follow-up in both groups (Supplemental Figure 1(B)). Titers of anti-Sm (*n* = 2), anti-RNP (*n* = 1) and anti-Ro (*n* = 1) antibodies declined over time. One patient with anti-Sm antibodies became negative during the first year of follow-up (Supplemental Figure 1(E)): this patient also became ANA negative.

Clinical and demographic features of the two groups of pSLE patients are shown in Table 1. We did not find statistically significant differences between the two groups. SLEDAI declined after the beginning of treatment and mostly remained under the threshold of four throughout the follow-up time with no differences between the two groups (Figure 1(e)) (Supplemental Figure 1(C)). Around 60% of SLE patients achieved Lupus low disease activity (LLDAS), whereas only 15% achieved remission according to the definition of remission in SLE (DORIS) criteria: in both cases this was due to glucocorticoid dosage above the defined thresholds. No significant differences in remission frequency were observed between the two groups (Table 1).

Taken together our data show that ANA titers decrease in patients with pSLE: 70% of patients remained positive, whereas 30% of patients became negative. Patients who achieved negative ANA did so in the first 6 months after the start of therapy. A good clinical response was observed in both groups.

### Interferon score in ANA positive and ANA negative patients with pSLE

We analyzed the levels of the interferon (IFN) score in the peripheral blood of patients with pSLE at 2 years of follow-up: no significant differences were found between the two groups. Since we reasoned that the numerosity of the samples could influence the result, we included six more patients for whom we had the ANA status at 2-year of follow-up, but not the serial data on ANA status every 4 months from diagnosis: two patients were ANA positive and four patients were ANA negative at 2 years of follow-up. The treatment regimen was similar to the first cohort, except that five patients were treated with cyclophosphamide at diagnosis and then started on mycophenolate as maintenance (Table 2). The IFN score was significantly higher in patients in Group 1 than in Group 0 (Mann Whitney test, *p* = .0026) (Figure 1(f)). We analyzed the clinical and laboratory features of the two groups of patients and we observed no significant differences (Table 2).

**Table 2.** Demographic and clinical characteristics at diagnosis and therapy at 2-years follow up of 21 patients with pSLE divided based on ANA positivity at 2-years follow up (ANA negative, Group 0; ANA positive, Group 1).

	All *n* = 21	Group 0 *n* = 8	Group 1 *n* = 13	*p*-value
Age at diagnosis, years [median, IQR]	13.00 [12.24, 15.00]	14 [12.33, 15.25]	12.63 [12.17, 14.00]	0.469
Female patients, *n* (%)	18 (85)	1 (25.0)	12 (92.3)	0.647
SLEDAI-2K at diagnosis [median, IQR]	11.00 [9.00, 14.00]	12.00 [10.75, 14.75]	10.00 [9.00, 12.00	0.135
SLEDAI-2K at follow-up [median, IQR]	0.00 [0.00, 2.00]	0.00 [0.00, 0.00]	2.00 [0.00, 2.00]	0.007
LLDAS at follow-up, *n* (%)	12 (57)	5 (63)	7 (54)	1.000
DORIS remission at follow-up, *n* (%)	2 (10)	1 (13)	1 (8)	1.000
C3, mg/dL [median, IQR]	54.00 [39.00, 66.25]	45 [40.00, 60.00]	60.00 [39.00, 67.50]	0.820
C4, mg/dL [median, IQR]	4.00 [2.00, 7.00]	3 [2.00, 7.00]	4.00 [3.00, 6.50]	0.909
Autoantibody profile				
anti-Sm+, *n* (%)	4 (19.0)	1 (12.5)	3 (23.1)	0.978
anti-RNP+, *n* (%)	2 (9.5)	0 (0.0)	2 (15.4)	0.688
anti-Ro+, *n* (%)	2 (9.5)	1 (12.5)	1 (7.7)	1.000
anti-La+, *n* (%)	0 (0.0)	0 (0.0)	0 (0.0)	NA
LAC, *n* (%)	2 (9.5)	1 (12.5)	1 (7.7)	1.000
anti-beta2GPI IgM+, *n* (%)	2 (9.5)	1 (12.5)	1 (7.7)	1.000
anti-beta2GPI IgG+, *n* (%)	2 (9.5)	1 (12.5)	1 (7.7)	1.000
anti-Cardiolipin IgM+, *n* (%)	2 (9.5)	1 (12.5)	1 (7.7)	1.000
anti-Cardiolipin IgG+, *n* (%)	2 (9.5)	1 (12.5)	1 (7.7)	1.000
anti-dsDNA + at diagnosis, *n* (%)	16 (76.1)	5 (62.5)	11 (84.6)	0.325
anti-dsDNA + at follow-up, *n* (%)	8 (38.1)	1 (12.5)	7 (53.8)	0.085
Clinical features				
Mucocutaneous, *n* (%)	12 (57.1)	5 (62.5)	7 (53.8)	1.000
Serositis, *n* (%)	3 (14.3)	1 (12.5)	2 (15.4)	1.000
Heamatological, *n* (%)	18 (85.7)	6 (75.0)	12 (92.3)	0.647
Musculoskeletal, *n* (%)	11 (52.4)	6 (75.0)	65 (38.5)	0.239
Constitutional, *n* (%)	16 (76.2)	6 (75.0)	10 (76.9)	1.000
Lupus nephritis, *n* (%)^a^	8 (38.1)	5 (62.5)	3 (23.1)	0.179
Medications during 2-year follow-up				
Glucocorticoids, *n* (%)	19 (90.5)	6 (75.0)	13 (100.0)	0.259
Glucocorticoid dose, mg/die	7.5 [6.2, 10.0]	7.5 [7.5, 9.4]	7.5 [5.0, 10.0]	0.783
Hydroxychloroquine, *n* (%)	20 (95.2)	8 (100.0)	12 (92.3)	1.000
Mycophenolate mofetil, *n* (%)	21 (100.0)	8 (100.0)	13 (100.0)	NA
Rituximab, *n* (%)	3 (14.3)	1 (12.5)	2 (15.4)	1.000
Cyclophosphamide, *n* (%)^b^	5 (23.8)	4 (50.0)	1 (7.7)	0.100

^a^Six patients with LN had a class III/IV nephritis, one had class II, and one class V.

^b^Five patients received cyclophosphamide at disease onset and then were on maintenance therapy with mycophenolate.

On the whole, patients with negative ANA at 2 years of follow-up showed decreased levels of the IFN score compared to ANA positive patients, suggesting that the disappearance of ANA is associated with reduced biologic activity of the IFN pathway compared to patients who remained ANA positive at 2 years of follow-up.

### Autoantibodies in children with pSS

**Figure 2.**
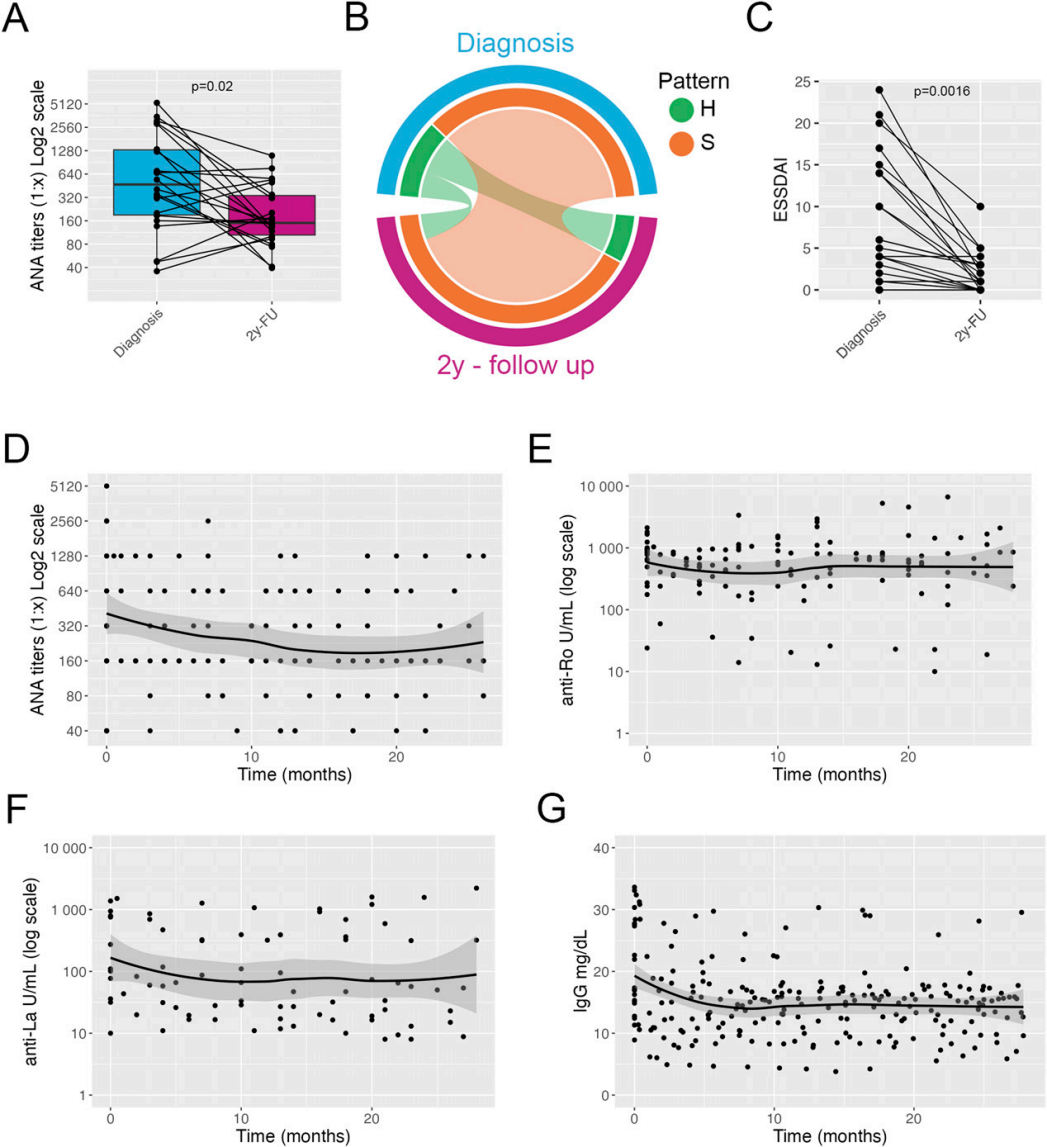
Autoantibody profile in patients with pSS. Titers of ANA at diagnosis and at 2-years follow-up; connected dots indicate individual patients (a). Circos plot showing the frequency of ANA pattern at diagnosis and at 2-years follow-up (b). Plot showing ESSDAI score at diagnosis and after 2 years; connected dots indicate individual patients (c). Plot showing the ANA titers at diagnosis and over a 2-years follow-up time; line indicate average trend with shaded confidence intervals (d). Plot showing the anti-Ro autoantibody titers at diagnosis and over a 2-years follow-up time; line indicate average trend with shaded confidence intervals (e). Plot showing the anti-La autoantibody titers at diagnosis and over a 2-years follow-up time; line indicate average trend with shaded confidence intervals (f). Plot showing serum levels of total IgG at diagnosis and over a 2-years follow-up time; line indicate average trend with shaded confidence intervals (g). A and C, Wilcoxon test. H = homogenous pattern, S = speckled pattern.

Twenty-two children with pSS were included in the study. The demographic, clinical and laboratory features of patients with pSS are shown in Table 3. At diagnosis 19 out of 22 patients were ANA positive with a titer equal to or higher than 1:80; the remaining three patients had positive ANA at a titer of 1:40. All patients had positive anti-Ro autoantibodies at disease onset. Fourteen (63%) patients were anti-La positive. After 2 years of follow-up, all patients remained ANA positive. Overall, the ANA titers were reduced at follow-up (Figure 2(a)) (Wilcoxon test, *p* = .02). Interestingly, in five patients we observed an increase in the titers of ANA: in the three patients with ANA at a titer of 1:40 at diagnosis, the titers increased to 1:160 for one patient and to 1:80 in two patients; in addition, in two patients, ANA titers increased to 1:640. Five patients (22%) showed a homogenous pattern, 17 patients (77%) showed a speckled pattern. The ANA pattern remained stable during the follow-up time except in two patients with a homogenous ANA pattern that converted to a speckled pattern during follow-up (Figure 2(b)). The ESSDAI score decreased significantly from diagnosis after 2 years of therapy (Wilcoxon test, *p* = .0016) (Figure 2(c)).

**Table 3.** Demographic and clinical characteristics of children with pSS at diagnosis.

	All *n* = 22
Age at diagnosis, years [median, IQR]	15.55 [13.29, 16.61]
Female sex, *n* (%)	19 (86.0)
ESSDAI [median, IQR]	4.00 [1.00, 13.00]
Autoantibody profile	
anti-Ro, *n* (%)	22 (100.0)
anti-La, *n* (%)	14 (63.0)
Laboratory parameters	
C3, mg/dL [median, IQR]	1.06 [0.94, 1.22]
C4, mg/dL [median, IQR]	0.16 [0.14, 0.23]
Serum IgG, g/L [median, IQR]	17.20 [13.35, 27.55]
Clinical features	
Xerostomia, *n* (%)	4 (18.2)
Xerophthalmia, *n* (%)	6 (27.3)
Heamatological, *n* (%)	10 (45.5)
Arthritis, *n* (%)	3 (13.6)
Constitutional, *n* (%)	9 (40.9)
Recurrent parotitis, *n* (%)	3 (20.0)
Salivary gland abnormalities in ultrasound, *n* (%)	14 (63.6)
Abnormal tear breakup time (TBUT), *n* (%)	7 (31.8)
Medications during 2-year follow-up	
Glucocorticoids, *n* (%)	8 (36.3)
Hydroxychloroquine, *n* (%)^a^	20 (90.1)
Mycophenolate mofetil, *n* (%)^b^	6 (27.3)
Methotrexate, *n* (%)	13 (59.1)
Rituximab, *n* (%)	2 (9.1)

^a^Two patients discontinued hydroxychloroquine during follow-up.

^b^Two patients switched from methotrexate (*n* = 1) and azathioprine (*n* = 1) to mycophenolate mofetil due to partial clinical response with methotrexate.

Titers of anti-Ro and anti-La antibodies did not change over time and no patient became negative for either autoantibody during follow-up (Figure 2(e), and (f)) (Supplemental Figure 2(D)) (anti-Ro: Wilcoxon test, *p* = .78; anti-La: Wilcoxon test, *p* = .38). Levels of IgG decreased in the first 6 months after diagnosis and starting of treatment, and remained stable thereafter (Figure 2(g)), with nine patients (40%) maintaining IgG levels above 16 g/L. Levels of complement fractions C3 and C4 remained within normal range throughout the follow-up time (data not shown). Overall, patients with pSS showed a good response to treatment and a decline in serum levels of total IgG and ANA titers. ANA pattern remained stable over time. Titers of anti-Ro and anti-La autoantibodies remained stable during the 2 years of follow up.

## Discussion

This study evaluated the evolution of autoantibodies in children diagnosed with pSLE and pSS over a 2-year period, underlining the dynamics of autoantibody profiles in response to disease treatment and its potential impact on clinical outcomes. In our cohort of children with pSLE, all patients at diagnosis exhibited high titers of ANA, which notably decreased over the 2-years follow-up. While 73% of patients remained ANA positive, 27% transitioned to an ANA-negative status, primarily within the first 6 months post-treatment initiation. Despite the decline in ANA titers, no significant differences were observed in the clinical and demographic features between the two groups and also in changes in anti-dsDNA antibodies and complement levels. However, ANA positive patients tended to be likely to be anti-dsDNA positive at 2-years follow-up. Interestingly, a notable difference was observed in the IFN score levels, with higher levels in ANA-positive patients at the end of the follow-up, suggesting that distinct molecular inflammatory pathways are associated with the persistence of ANA. In children with pSS, we observed that all patients remained ANA positive throughout follow-up, albeit with a general reduction in ANA titers. The persistence of anti-Ro and anti-La antibodies underscored the continuous autoantibody production in pSS. Despite this, a favorable clinical response was evident from the decreased ESSDAI scores and stable hypergammaglobulinemia, highlighting the efficacy of treatment in managing clinical disease, but without completely reversing the biological substrates of autoimmunity.

The production of autoantibodies is central in autoimmune connective tissue diseases, as SLE and SS.^9^ Autoreactive B and T cell are routinely generated during lymphocyte maturation and several mechanisms have evolved to silence these autoreactive clones and prevent autoimmunity.^10^ However, autoreactivity exerts also some positive effects on immune functions, hence, the elimination of autoreactive clones is not so drastic and low levels of autoreactivity are usually favored.^11,10^ In SLE several evidence point to defective tolerance mechanisms at different stages of B cell maturation.^12–14^ Before and during a flare, activated naïve B cells differentiate into plasmablasts secreting antibodies, including autoantibodies (i.e. anti-dsDNA).^13,15,16^

It has been demonstrated that years before the occurrence of full-blown SLE, the titers of autoreactive antibodies slowly increase,^17^ together with different cytokines, such as interferons.^18^ Less is known about the kinetics of ANA and autoantibodies in SLE after disease onset. Several groups have shown that the titers of ANA decreased with time, and around 8%–24% of SLE patients seroconverted to ANA negative.^19–22^ Our findings confirm the same trend in children with pSLE: for all patients ANA titers decreased significantly and, in 27% of patients, ANA became negative. Interestingly, as we could analyze the titers of ANA every 4 months from diagnosis, we observed that the patients who became ANA negative, did so in the first 6 months of follow-up. We also observed lower levels of the interferon signature in patients who became ANA negative compared to patients who did not, indicating that autoantibody negativization is associated with a different cytokine milieu. We showed that almost all patients with pSLE at disease onset had an increased type I and type II interferon score, highlighting the generalized activation of these pathways at time of onset of pSLE.^23^ Thus, we hypothesize that in patients who became ANA negative, the activation of the interferon pathways abated together with the production of autoantibodies.

In patients with pSS, we observed a decrease in the titers of ANA during follow-up; however, no patient became ANA negative, and the ANA pattern remained fairly stable. Interestingly, in a small number of patients with low levels of ANA at diagnosis, ANA titers increased during follow-up. These patients may have autoantibodies that bind only the Ro antigen and no other specificities and for this reason the ANA reactivity may be low or absent.^24^ Titers of anti-Ro and anti-La autoantibodies remined quite constant in our population of patients with pSS, confirming previous literature.^3,25^ Interestingly, titers of anti-ENA antibodies of patients with pSLE declined over time and in one patient became negative. Although the small sample size, this evidence points to a different pathway of autoreactive B cell activation in the two autoimmune diseases. Levels of hypergammaglobulinemia in patients with pSS decreased soon after diagnosis and start of treatment; however, levels remained above the threshold of 16 g/L in around 40% of patients with pSS during follow-up. Thus, while ANA titers changed over time, indicating a modulation in the production of some of the ANA specificities, the stability in titers of anti-Ro and anti-La autoantibodies may reflect the production of these autoantibodies from a different compartment. We would speculate that some of the ANA can be produced by plasmablasts derived from activated B cells, whereas anti-Ro and anti-La autoantibodies may come from long-live plasma cells in pSS. We have shown that B cells have an activated phenotype in patients with pSS, with the expansion of atypical memory B cells and T peripheral helper (T_PH_) cells and an altered tolerance checkpoint at the differentiation into plasmablasts (i.e. more autoreactive B cells are allowed to be activated and to differentiate into antibody-secreting cells).^26^ Taken together our results point to an ongoing B cell activation state in patients with pSS, that is not reversed by current treatment, as shown by the persistent B cell abnormalities^26^ and the production of total IgG and autoantibodies, despite clinical improvement.

Among the many autoantibodies, anti-dsDNA is the only antibody considered a marker of disease activity, thus anti-dsDNA titers monitoring is a useful tool in SLE assessment. By contrast, a single ENA antibody determination at first evaluation is thought to suffice for clinical purposes due to evidence showing little variations in ENA levels over time.^3,4,25^ However, recent evidence suggests that in a subset of patients with SLE both ANA and ENA levels may decrease over time secondary to the natural history of the disease or to medication exposure.^3,21^ Additionally, ANA production reflects the activation of B cells and different autoantibodies may be produced by different B cell subsets as already mentioned: for example, the transient production of anti-dsDNA autoantibodies can be driven by short-lived plasmablasts,^15^ whereas ENA antibodies, recognizing protein antigens, can be secreted by long-lived plasma cells. Thus, several factors may account for the different kinetics of autoantibodies during the course of SLE. Only a handful of longitudinal studies has been conducted to analyze ANA and ENA changes over time in different autoimmune diseases,^3,19,21,27–29^ and further work is required to investigate in larger cohorts how autoantibodies change with disease course and if these changes reflect different underlying biological pathways.

One of the limitations of our study is the small number of patients with pSLE and pSS, which is mostly explained by the rarity of these diseases among children. This is balanced by the fact that samples were collected at the time of disease onset and before therapy was initiated. Indeed, a major strength of our study is the enrollment of a relatively homogeneous cohort of patients with high disease activity at diagnosis, all before the start of treatment. Additionally, all patients were treated with a similar immunosuppressive regimen, allowing us to evaluate the impact of treatment on the antibody profile in a clean, homogeneous, and well-characterized cohort. Another limitation is the relatively short time of follow-up of 2 years, compared to other studies with 5 to 10 years of follow-up.^19–21^ All studies suggest a reduction in ANA titers over time, and the change in autoantibodies titers we observed could be even greater with a longer follow-up. Future multicentric prospective studies will be needed to confirm our results and investigate in depth the clinical implications.

Our results indicating a fluctuation in autoantibodies may have repercussions in disease classification, management and development of new therapies. Positive ANA is an entry criterion for the 2019 EULAR/ACR classification criteria for SLE; thus, the fact that up to 30% of patients may become ANA negative soon after treatment initiation may affect how we classify patients. More importantly, ANA status may also reflect a different response to therapies that target B cells, making it a possible marker for patient stratification.

Collectively, these findings underscore the complexity of autoantibody dynamics in pediatric autoimmune diseases and their potential as diagnostic markers, indicators of disease activity and predictors of treatment response. The study insights into the fluctuating patterns of autoantibodies in pSLE and pSS offer a promising avenue for tailoring patient-specific management strategies, ultimately aiming to improve long-term outcomes in these diseases.

## Supplemental Material

Supplemental Material - Different patterns of longitudinal changes in antinuclear antibodies titers in children with systemic lupus erythematosus and sjogren syndrome Supplemental Material for Different patterns of longitudinal changes in antinuclear antibodies titers in children with systemic lupus erythematosus and sjogren syndrome by Patricia Morán Álvarez, Claudia Bracaglia, Rebecca Nicolai, Luigi Giovannelli, Ivan Caiello, Alessandra Boni, Valentina Matteo, Gian Marco Moneta, Virginia Messia, Fabrizio De Benedetti and Emiliano Marasco in Lupus.
