# Supplementary material for: Different patterns of longitudinal changes in antinuclear antibodies titers in children with systemic lupus erythematosus and Sjögren's syndrome
Source: Lupus. 2024 Nov 4;33(14):1594–604. doi: 10.1177/09612033241298729 (PMC11613521; doi:10.1177/09612033241298729)
Supplement: Supplemental Material - Different patterns of longitudinal changes in antinuclear antibodies titers in children with systemic lupus erythematosus and sjogren syndrome [file sj-pdf-1-lup-10.1177_09612033241298729.pdf]

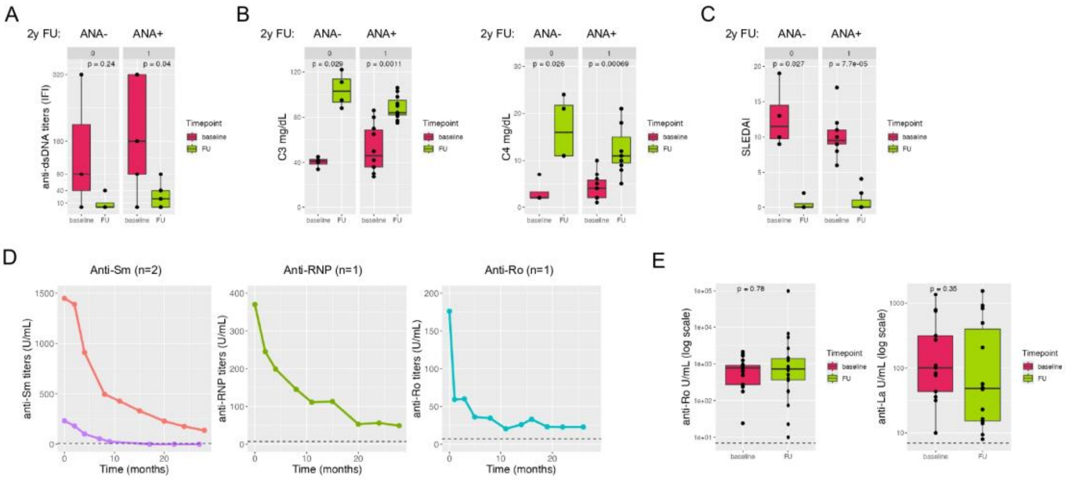

Supplementary Figure 1

Boxplots showing titers of anti-dsDNA antibodies at diagnosis (baseline) and at 2-year follow-up (FU); pSLE patients are divided in two groups according to ANA status at 2-year FU (A). Boxplots showing levels of complement fractions C3 and C4 at diagnosis (baseline) and at 2-year FU; pSLE patients are divided in two groups according to ANA status at 2-year FU (B). Boxplots showing SLEDAI scores at diagnosis (baseline) and at 2-year FU; pSLE patients are divided in two groups according to ANA status at 2-year FU (C). Plots showing the titers of anti-Sm, anti-RNP and anti-Ro antibodies in 4 patients with SLE; dashed line indicates cut-off value (D). Boxplots showing titers of anti-Ro and anti-La antibodies at diagnosis (baseline) and at 2-year FU; dashed line indicates cut-off value (E).
